# Comparative effects of replacing soybean meal in feeds with processed soybean meal on intestinal health and growth of nursery pigs when fed pharmacological level of zinc

**DOI:** 10.5713/ab.24.0767

**Published:** 2025-02-27

**Authors:** Lan Zheng, Jung Yeol Sung, Sung Woo Kim

**Affiliations:** 1 Department of Animal Science, North Carolina State University, Raleigh, NC, USA

**Keywords:** Intestinal Health, Pig, Processed Soybean Meal, Zinc

## Abstract

**Objective:**

The objective was to evaluate the effects of partially replacing soybean meal (SBM) in nursery pig diets with enzyme-treated soybean meal (ESBM), fermented soybean meal (FSBM), or fermented soybean meal containing probiotic microorganism (PFSBM) on jejunal mucosa-associated microbiota, immune responses, intestinal morphology, and growth performance of nursery pigs.

**Methods:**

Forty-eight weaned pigs (initial body weight = 7.8 ± 0.7 kg) were randomly allocated to four dietary treatments in a randomized complete block design and fed for 25 d in three phases (5, 10, and 12 d, respectively). Dietary treatments were corn-SBM-based diet (SBM diet) and the SBM diet in which 70 g/kg of SBM was replaced with ESBM (ESBM diet), FSBM (FSBM diet), or PFSBM (PFSBM diet). Zinc oxide was supplemented at 2.5 g/kg (2,000 mg/kg of zinc) in the experimental diets for phases 1 and 2. Pigs were housed individually in pens (1.50 m×0.74 m) equipped with a feeder and a nipple drinker. Serum was collected on d 24 and pigs were euthanized on d 27 to collect tissues and mucosa in the jejunum.

**Results:**

The PFSBM diet increased (p<0.05) the relative abundance of *Lactobacillus*, whereas it tended to decrease (p = 0.072) the abundance of *Pseudomonas* compared with the SBM diet. The ESBM diet increased (p<0.05) gain to feed ratio from d 5 to 15 compared with the SBM diet. However, partially replacing SBM at 70 g/kg with ESBM, FSBM, or PFSBM did not affect immune responses in serum and jejunal mucosa, intestinal morphology in the jejunum, and overall growth performance of nursery pigs.

**Conclusion:**

Partially replacing SBM with various processed SBM did not affect immune responses, intestinal morphology, and overall growth performance when diets were supplemented with zinc at pharmacological level in early phases.

## INTRODUCTION

Soybean meal (SBM) is a commonly used protein supplement in pig feeds across all stages of pig growth. However, SBM contains various anti-nutritional compounds, with the most significant being trypsin inhibitors, oligosaccharides, and allergenic proteins [1]. Trypsin inhibitors can bind to trypsin or chymotrypsin and make them inactive, which can decrease ileal amino acid digestibility [2]. Undigested oligosaccharides in SBM ferment in the hindgut and cause flatulence and diarrhea because pigs lack endogenous α-galactosidase to digest them [3]. Globulins are the primary form of protein storage in soybeans and contain allergenic proteins (e.g., glycinin and β-conglycinin), which cause inflammation in the intestinal tract of pigs fed a large amount of SBM [4–6].

Weaning is the most stressful event in a pig’s life due to the transition from sow’s milk to solid feed, transportation to separate facilities, and fierce competition for social hierarchy [7]. Post-weaning stress is associated with disrupted intestinal microbiota, compromised mucosal immune function, and damaged intestinal tissues, which leads to reduced digestion, nutrient absorption, and growth performance [8]. Due to their immature intestinal tract, the impact of anti-nutritional compounds in SBM is more pronounced in weaned pigs compared with mature pigs. Particularly, weaned pigs are hypersensitive to allergenic proteins in SBM, which can potentially induce inflammation and reduce their growth performance [5]. For this reason, supplementing SBM in nursery diets can be challenging although it is the major protein supplement providing amino acids to pigs [9].

Processing SBM with enzymes or through fermentation is a way to address the issue associated with SBM [10]. Enzyme-treated soybean meal (ESBM) and fermented soybean meal (FSBM) refer to SBM that has been processed with enzyme cocktails or fermented by beneficial bacteria, respectively [11]. The superiority of ESBM and FSBM over SBM is because of the hydrolysis of anti-nutritional compounds in SBM achieved either through enzymes directly supplemented or produced by microorganism during fermentation. Furthermore, an additional heating process is required during the processing ESBM and FSBM, which can potentially inactivate heat-labile anti-nutritional compounds. For these reasons, the concentration of anti-nutritional compounds in ESBM and FSBM is lower compared with SBM [12,13]. In addition to hydrolyzing anti-nutritional compounds, the enzymes also break down proteins into smaller peptides, enhancing digestion and absorption [14]. Therefore, partially replacing SBM with ESBM or FSBM in nursery diets could potentially improve the intestinal health and growth of nursery pigs [14–17].

Pharmacological levels of zinc have been widely used in nursery diets to suppress diarrhea and maximize growth performance of pigs [18]. It has been banned in European countries in 2022 due to concerns about antimicrobial resistance [19], whereas the supplementation of zinc oxide to nursery diets in excess of zinc requirements is a common practice in the United States [20]. The current study aimed to test the hypothesis that partially replacing SBM with ESBM or FSBM enhances intestinal health and growth in nursery pigs fed diets with pharmacological level of zinc in early phases. The mechanism for improving intestinal health and growth by partially replacing SBM with the processed SBM is based on reducing anti-nutritional compounds, primarily allergenic proteins. To test the hypothesis, the objective of this study was to evaluate the effects of partially replacing SBM in nursery pig diets with different types of processed SBM on jejunal mucosa-associated microbiota, immune responses, intestinal morphology, and growth performance of nursery pigs when fed diets containing zinc oxide.

## MATERIALS AND METHODS

The protocol of this experiment was reviewed and approved by North Carolina State University Animal Care and Use Committee (Raleigh, NC, USA).

### Test ingredients

The ESBM was a commercially available product (Hamlet Protein, Horsens, Denmark) that is enzymatically hydrolyzed using a proprietary enzyme blend (Tables 1, 2). The FSBM and fermented soybean meal containing probiotic microorganism (PFSBM) were commercially available products (CJ CheilJedang Co., Seoul, Korea) processed through fermentation using *Bacillus amyloliquefaciens*. The difference in the processing between FSBM and PFSBM is that PFSBM contained active microorganisms used for fermentation (*Bacillus amyloliquefaciens* at 1.9×10^7^ colony forming units [CFU]/g) whereas the microorganisms in FSBM were inactived by heating.

### Molecular weight distribution of peptides in test ingredients

The molecular weight distribution of peptides in SBM, ESBM, FSBM, and PFSBM was assessed using an analytical ultracentrifuge (ProteomeLab XL-A; Beckman Coulter, Fullerton, CA, USA) following the procedure outlined by Schuck et al [21]. Briefly, homogenized samples were centrifuged at 12,300×g for 15 min at 4°C using an Eppendorf MiniSpin (Eppendorf AG, Hamburg, Germany). A total of 200 scans were collected at 6-min intervals. Proteins were prepared with 2 mL of 20 mM Tris-HCl buffer containing 0.1% sodium dodecyl sulfate and 5 mM dithiothreitol at pH 7.4. Aliquots (110 μL) of the sample solution were loaded into 6-sector sample cells. Absorbance was monitored at 280 nm for the loaded samples. Sedimentation velocity data were analyzed using the SEDFIT software (version 11.8; National Institutes of Health, Bethesda, MD, USA) to generate the sedimentation coefficient distribution for the protein samples.

### Animals, experimental design, and experimental diets

Forty-eight weaned pigs (24 barrows and 24 gilts) with an initial body weight of 7.8±0.7 kg were randomly allocated to four dietary treatments in a randomized complete block design, using initial body weight and sex as blocks. Pigs were housed individually in pens (1.50 m×0.74 m) equipped with a feeder and a nipple drinker, allowing them free access to both feed and water. All pens were located in a single room, which was mechanically ventilated, with the temperature maintained between 27°C and 29°C. The four diets included a corn-SBM-based diet (SBM diet) and the SBM diet in which 70 g/kg of SBM was replaced with ESBM (ESBM diet), FSBM (FSBM diet), or PFSBM (PFSBM diet; Tables 3, 4). The inclusion rate of the processed SBM was determined based on previous studies [14,22]. Zinc oxide was supplemented at 2.5 g/kg (2,000 mg/kg of zinc) in the experimental diets for phases 1 and 2. Pigs were fed for 27 d (phase 1: d 0 to 5, phase 2: d 5 to 15, and phase 3: d 15 to 27). Each diet was formulated to meet or exceed the nutrient requirements for nursery pigs provided by NRC [23].

### Sample and data collection

Body weight of pigs and feed disappearance of each pen were measured on d 0, 5, 15, and 27 to determine average daily gain, average daily feed intake, and gain to feed ratio. On d 24 at 8 AM, blood samples were collected from the jugular vein of all pigs using BD Vacutainers (Franklin Lakes, NJ, USA). The collected samples were then centrifuged at 3,000×g for 15 min at 4°C to obtain serum. The serum samples were stored at −80°C before the analysis. On d 27, all pigs were euthanized using a captive bolt followed by exsanguination. Subsequently, 20 cm of mid-jejunal sections located 3 to 4 m beyond the pyloric valve of the stomach were collected and rinsed with a 0.9% saline solution. The mucosa were scraped from the first 15 cm of the collected jejunum, then immediately frozen in liquid nitrogen and stored at −80°C before the analysis. The remaining 5 cm was fixed in a 10% buffered formalin solution.

### Relative abundance of the mucosa-associated microbiota in the jejunum

The QIAamp DNA Stool Mini Kit (#51504; Qiagen, Germantown, MD, USA) was used to extract DNA from the collected jejunal mucosa. The extracted DNA samples were sent to MAKO Medical Laboratories (Raleigh, NC, USA) for quantitative polymerase chain reaction analysis of 16S rDNA sequences. The Ion Chef instrument and Ion S5 system were used to prepare samples for template sequencing, respectively. The Ion 16S Metagenomics Kit (A26216; ThermoFisher Scientific Inc. Wilmington, DE, USA) was utilized to amplify the variable regions (V2, V3, V4, V6, V7, V8, and V9) of the 16S rRNA gene. The Ion Xpress Plus Fragment Library kit (Cat. 4471269; ThermoFisher Scientific Inc.) was employed to create libraries from the amplified regions, along with the Ion Code Barcode Adapters 1-384 kit (A29751; ThermoFisher Scientific Inc.). The Ion Universal Library Quantitation kit (A26217; ThermoFisher Scientific Inc.) was utilized to quantify the libraries. The Torrent Suite Software (version 5.2.2) was employed to process the sequences, generating unaligned bam files for subsequent analysis. The Ion Reporter Software Suite (version 5.2), a set of bioinformatics analysis tools, was utilized for sequence data analysis, alignment with the GreenGenes and MicroSeq databases, as well as for generating alpha and beta diversity plots and operational taxonomic unit (OTU) tables. All samples exhibited sequencing coverage depth exceeding 1,000×. The OTU data were converted to relative abundance for subsequent statistical analysis, and OTU data with an abundance of less than 0.05% at each level were grouped as “others.”

### Immune responses in the jejunum

One gram of the collected jejunal mucosa was homogenized using a homogenizer (Tissuemiser; Thermo Fisher Scientific Inc., Waltham, MA, USA) to analyze its immunoglobulin A (IgA), immunoglobulin G (IgG), tumor necrosis factor (TNF)-α, and interleukin (IL)-6. The supernatant was collected after centrifuging the homogenized mucosa at 14,000×g for 30 min at 4°C. The total protein content of the supernatant was measured using the pierce bicinchoninic acid protein assay kit (23225#; Thermo Fisher Scientific Inc.) following the procedure described by Deng et al [10]. The measured total protein concentration was used to standardize the concentration of the immune response criteria. The jejunal mucosa was analyzed using enzyme-linked immunosorbent assay (ELISA) kits for pig IgA (E101–102) and pig IgG (E101–104) from Bethyl Laboratories, Inc. (Montgomery, TX, USA), respectively. The contents of TNF-α and IL-6 in jejunal mucosa were measured using ELISA kits (R&D Systems, Minneapolis, MN, USA) following the method described by Jang and Kim [24]. The serum concentrations of IgG, TNF-α, and IL-6 were also analyzed using the same methods used for the jejunal mucosa.

### Intestinal morphology in the jejunum

The collected jejunal tissues were used to evaluate intestinal morphology. After 48 h of fixation in 10% buffered formaldehyde, two sections of the tissues (approximately 2 mm each) were cut, placed in a cassette, and transferred to a 70% ethanol solution. The processed samples were then sent to the North Carolina State University Histology Laboratory (College of Veterinary Medicine, Raleigh, NC, USA) for dehydration, embedding, and staining.

The microscope Olympus CX31 (Lumenera Corporation, Ottawa, Canada) and software (Infinity 2–2 digital CCD) were used to measure villus height and crypt depth at a magnification of 40× following the procedure described by Deng et al [10]. Ten intact villi and crypts were selected to represent the intestinal morphology for each pig. Villus length was measured from the tip to its junction with the crypt, whereas crypt depth was measured from this junction to the base. The ratio of villus height to crypt depth was calculated by dividing villus height by crypt depth.

### Chemical analysis

The SBM, ESBM, FSBM, and PFSBM were analyzed for dry matter (method 930.15) and crude protein (method 990.03) as described in AOAC [25]. The experimental diets were also analyzed for crude protein using the same method. The concentrations of amino acids in the ingredients were measured using ion-exchange chromatography coupled with post-column derivatization using ninhydrin. Prior to analysis, samples were released from the protein through hydrolysis with 6 N HCl for 24 h at 110°C (method 982.30; AOAC [25]). Methionine and cystine were assessed as methionine sulfone and cysteic acid, respectively, following overnight cold performic acid oxidation before hydrolysis. Tryptophan was evaluated after being hydrolyzed with NaOH for 22 h at 110°C.

### Statistical analysis

Homogeneity of the variances and normality of data were confirmed using the UNIVARIATE of SAS (SAS Institute Inc., Cary, NC, USA). Data were analyzed using the MIXED procedure of SAS. The model included dietary treatment as a fixed effect, whereas initial body weight and sex as random effects. The number of replications per treatment was pre-determined based on a power test with the following condition: To determine the significance difference in expected means at 15% at p<0.05, considering 12.5% coefficient of variation based on previous studies with pigs of similar genetic background and under similar research environment [26,27], and with a desired power of test (1 – beta) set at 95%, the power analysis indicated an 80% power. Based on the analysis, the minimum replications required for treatment was 12 [28]. Least square means for each treatment were calculated, and differences among least squares means were tested using the PDIFF option with Tukey’s adjustment. An experimental unit was a pen. Significance and tendency were declared at p<0.05 and 0.05≤p<0.10, respectively.

## RESULTS

The population of *Bacillus amyloliquefaciens* was greater in PFSBM (1.9×10^7^ CFU/g) compared with FSBM (1.1×10^2^ CFU/g; Table 1). The molecular weight distribution of peptides in each ingredient consists of two or three fractions (Table 2). The greatest molecular weight fraction of peptides was observed in SBM (134,000 Da), followed by ESBM (61,015 Da), FSBM (10,750 Da), and PFSBM (7,870 Da). The average molecular weight of peptides was the greatest in SBM (34,300 Da), followed by ESBM (13,747 Da), PFSBM (6,057 Da), and FSBM (2,816 Da).

**Figure 1:**
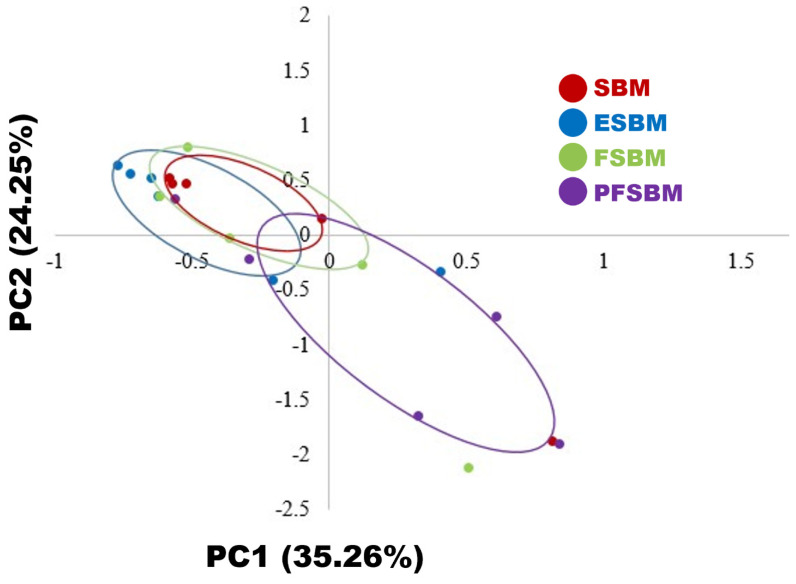
Principal component analysis of jejunal mucosal microbiota of nursery pigs fed diets containing soybean meal (SBM), enzyme-treated soybean meal (ESBM), fermented soybean meal (FSBM), or fermented soybean meal containing probiotic microorganism (PFSBM). Red: SBM, Blue: ESBM, Green: FSBM, Purple: PFSBM. The microbial community of pigs fed the PFSBM diet was different (p<0.05) from the other diets, indicating that the microbial community was shifted by the PFSBM diet.

The pigs remained healthy throughout the experiment, and there was no missing data. Differences in alpha diversity metrics in jejunal-associated mucosa were not observed (Table 5). According to the principal component analysis, the overall microbial community of jejunal-associated microbiota of the PFSBM diet was different (p<0.05) from the other diets (Figure 1). At the phylum level, the relative abundance of microbiota in the jejunal mucosa was not affected by the dietary treatments (Table 6). At the family level, The PFSBM diet increased (p<0.05) the relative abundance of *Lactobacillaceae* compared with the other diets (Table 7). At the genus level, the PFSBM diet increased the relative abundance of *Lactobacillus* compared with the other diets and decreased the abundance of *Stenotrophomonas* compared with the ESBM diet (p<0.05; Table 8). The PFSBM diet tended to decrease (p = 0.072) the relative abundance of *Pseudomonas* compared with the SBM and FSBM diets. The ESBM diet tended to decrease (p = 0.063) serum TNF-α concentration compared with the PFSBM diet (Table 9). There was no significant difference in villus height, crypt depth, and the villus height to crypt depth ratio in the jejunum among the dietary treatments (Table 10). The ESBM diet increased (p<0.05) gain to feed ratio from d 5 to 15 compared with the SBM diet (Table 11).

## DISCUSSION

Soybean meal is not efficiently digested and absorbed by nursery pigs due to their immature intestinal tract and the presence of anti-nutritional compounds including glycinin and β-conglycinin in SBM although it is the major protein supplement providing amino acids in swine diets [1]. One way to address this issue is to partially replace SBM with either animal protein supplements (e.g., fish meal, meat and bone meal, plasma protein, and poultry meal), processed SBM (e.g., ESBM and FSBM), or both in nursery diets. Animal protein supplements lack anti-nutritional compounds and provide highly digestible protein with a balanced amino acid profile, whereas processed SBM contains a reduced amount of anti-nutritional compounds. For these reasons, both types of ingredients could be potentially superior over SBM. However, animal protein supplements are costly and may raise safety concerns [29]. Additionally, these animal protein supplements undergo a rendering process involving steam heating at high temperatures (115°C to 145°C for 40 to 90 min), which can reduce the quality of animal proteins and increase quality inconsistency [30,31]. For these reasons, animal protein supplements as well as SBM in nursery diets could be partially replaced by the processed SBM. In a recent publication, however, Deng et al [10] reported that partially replacing animal protein supplements with FSBM decreased growth performance and increased pro-inflammatory responses in the jejunum. This suggests that animal protein supplements are not just protein supplements; they also contain bioactive compounds that suppress inflammation in the intestines of pigs [32]. For this reason, animal protein supplements were added to the experimental diets in the current study. Moreover, zinc oxide was supplemented in the current diets for phases 1 and 2 (2,000 mg/kg of zinc) to prevent diarrhea and maximize growth performance of pigs during the first two phases, which is a common practice in the swine industry in the United States [20].

The PFSBM contained active microorganisms used for fermentation, whereas the microorganisms were inactived by heating in FSBM, resulting in a higher population of *Bacillus amyloliquefaciens* in PFSBM (1.9×10^7^ CFU/g) compared with FSBM (1.1×10^2^ CFU/g). This difference may have contributed to the higher crude protein concentration in PFSBM compared with FSBM (568 g/kg vs. 422 g/kg). However, the reason for the higher crude protein in PFSBM is speculated to be due to non-protein nitrogen as the amino acid profiles of FSBM and PFSBM are similar. Molecular weight distribution of peptides could be a potential indicator of glycinin and β-conglycinin. The molecular weight of glycinin ranges from 300,000 to 380,000 Da, with its acidic and basic subunits at 35,000 Da and 20,000 Da, respectively [33]. In contrast, β-conglycinin has a molecular weight of peptides ranging from 150,000 to 200,000 Da, with the α, α’, and β subunits at 72,000 Da, 68,000 Da, and 52,000 Da, respectively [34]. The molecular weight of peptides in ESBM, FSBM, and PFSBM was lower compared with SBM and rarely overlapped with the subunits of glycinin and β-conglycinin, indicating that these anti-nutritional compounds had been efficiently hydrolyzed. The lower concentrations of glycinin and β-conglycinin in processed SBM compared with SBM have been consistently reported in previous studies [10,12].

In the current study, partial replacing SBM with various processed SBM did not affect alpha diversity metrics of jejunal-mucosa associated microbiota (Chao1: richness, Shannon: richness and evenness, and Simpson: richness and evenness). However, the PFSBM diet altered the overall microbial community in the jejunal mucosa compared with the other diets, indicating that the microbial community was shifted by the PFSBM diet. To investigate details on the shift, relative abundance of the microbiota on genus level was compared. The distinct jejunal mucosa microbiota of pigs fed the PFSBM diet is partially attributed to the greater relative abundance of *Lactobacillus*, as corroborated by previous results [35,36]. *Bacillus amyloliquefaciens* is aerobic and rapidly consumes oxygen, which promotes the colonization of anaerobic bacteria, whereas inhibits aerobic bacteria [37]. Additionally, *Bacillus amyloliquefaciens* may lower the pH of the intestinal tract, suppressing the growth of pathogenic bacteria and promoting the colonization of beneficial anaerobic bacteria such as *Lactobacillus*. From this perspective, the relatively lower abundance of *Pseudomonas* in the PFSBM diet compared with the SBM diet may not be surprising because *Pseudomonas* is typically aerobic and pathogenic [38]. *Stenotrophomonas* is an undesirable microbe due to its ability to cause opportunistic infections and potentially reduce growth of pigs [39,40]. The relative abundance of *Stenotrophomonas* is the lowest numerically in the PFSBM diet, which can be partially attributed to inhibition by *Bacillus amyloliquefaciens*, as mentioned above. However, the greater relative abundance of *Stenotrophomonas* in the jejunal mucosa of the ESBM diet compared with the PFSBM diet remains unclear and has not been reported in the literature. Overall, it is speculated that the jejunal-mucosa associated microbiota of the PFSBM diet might be more beneficial to the host compared with the other diets, which requires experimental validation. Moreover, the population of *Bacillus amyloliquefaciens* in the FSBM diet [(1.1×10^2^ CFU/g)×0.7 = 7.7 CFU/g of diet] may not be sufficient to induce the positive effects associated with *Bacillus amyloliquefaciens* on mucosa-associated microbiota.

However, the shift in microbiota induced by *Bacillus amyloliquefaciens* did not lead to favorable changes in the other response criteria, indicating that an increase in potentially beneficial bacteria or a decrease in pathogenic bacteria do not always guarantee an improvement in phenotype. The pro-inflammatory cytokine, TNF-α, promotes inflammation both locally and systemically [8]. In the current study, the serum TNF-α concentration tended to be greater in pigs fed the PFBSM diet compared with the ESBM diet, which was not expected. The PFSBM might contain a higher concentration of allergenic proteins compared with ESBM, potentially triggering inflammation and increasing serum TNF-α. Although molecular weight of peptides was similar between PFSBM and ESBM in the current study, it may not always be strongly related to allergenic protein concentration. Cervantes-Pahm and Stein [12] reported a higher allergenic protein concentration in FSBM compared with ESBM despite their similar molecular peptide weight. A potential side effect of probiotic overdose might be another possible explanation. When the intestinal tract is exposed to excessive probiotics (potentially beneficial bacteria), the balance between beneficial and pathogenic bacteria is disrupted, leading to an imbalance in the microbial ecosystem and triggering systematic inflammatory pathways [41]. For example, supplementing probiotics (e.g., *Lactobacillus rhamnosus* and *Bacillus subtilis*) at doses exceeding the recommended levels increased TNF-α concentration in serum or decreased growth performance in nursery pigs, indicating the potential deterimental impact of probiotic overdose on nursery pigs [42–44]. The *Bacillus amyloliquefaciens* count in the PFSBM diet in the current study [(1.9×10^7^ CFU/g)×0.7 = 1.33×10^7^ CFU/g of diet] might be considered an overdose, but the threshold for overdose may vary depending on the species and strain of *Bacillus*.

However, the lack of change in TNF-α at the jejunal mucosa remains unclear. Mucosa serves as the first line of defense in the immune responses in the intestinal tract [8]. Changes in TNF-α concentration in the jejunal mucosa reflect local inflammation in response to dietary treatment, whereas changes in serum indicate systemic inflammation [45]. The tendency of increasing TNF-α was observed only in serum and the mechanisms for this remain unclear. It is speculated that *Bacillus amyloliquefaciens* may have indirectly caused systemic inflammation by affecting tissues other than the jejunum [46]. However, this speculation may not make sense because the majority of immune cells in the intestinal tract are located in the small intestine, and the jejunum is the longest part of the small intestine [47,48]. Furthermore, the lack of significant differences in jejunal or serum IgA, IgG (indicators of humoral immune status) [49,50], and IL-6 (which has both pro- and anti-inflammatory characteristics) [51,52] among the dietary treatments also does not support the idea that the PFSBM diet induced pronounced local and systemic inflammation compared with the ESBM diet.

In the current study, there was no difference in intestinal morphology indicators (villus height, crypt depth, and villus height to crypt depth ratio) among the dietary treatments. For growth performance, the gain to feed ratio from d 5 to 15 was greater in the ESBM diet compared with the SBM diet, which is attributed to lower levels of glycinin, β-conglycinin, oligosaccharides, and higher concentration of digestible amino acids [1,12]. Furthermore, the expected greater expression of tight junction proteins and lower intestinal permeability in the ESBM diet compared with the SBM diet might be another possibility because tight junction proteins could be damaged by increased apoptosis of enterocytes when pigs are fed high concentrations of glycinin or β-conglycinin [53–55]. Therefore, partially replacing SBM with ESBM at 70 g/kg from d 5 to 15 can potentially provide economic benefits for nursery pigs, even when fed diets containing pharmacological levels of zinc during the early phases, primarily due to the immature development of their intestinal tract.

In contrast, partially replacing SBM with FSBM or PFSBM did not influence growth performance, likely due to the higher concentrations of glycinin and β-conglycinin compared with ESBM, as consistently reported previously [10,12]. Furthermore, there was no difference in intestinal morphology and growth performance between the ESBM and PFSBM diets, indicating that the tendency for increase in serum TNF-α by the PFSBM diet might not be significant enough to affect other response criteria.

In summary, partially replacing SBM with ESBM, FSBM, or PFSBM did not affect immune responses, intestinal morphology, and growth performance (except for gain to feed during d 5 to 15 with ESBM), which contradicts findings from previous studies [16,56,57]. These results indicate that partially replacing SBM with processed SBM at 70 g/kg might not be economically beneficial during the overall nursery period when nursery pigs are fed diets containing pharmacological levels of zinc during the early phases. Two possible explanations may account for the discrepancy. First, the inclusion rate of SBM in the SBM diet (phase 1: 200 g/kg, phase 2: 230 g/kg, and phase 3: 280 g/kg) might not have been high enough to induce the negative impact of glycinin and β-conglycinin, or the rate of SBM replacement with the processed SBM at 70 g/kg might not have been sufficient, or both. For example, partially replacing SBM in diets containing 220 to 240 g/kg of SBM with ESBM or FSBM at 90 to 152 g/kg improved immune responses, intestinal morphology in the small intestine, and growth performance in early phases of nursery pigs [16,56,58,59]. However, this speculation may not be valid because replacing SBM with FSBM at 50 to 60 g/kg increased body weight gain and expression of tight junction proteins in the ileum of nursery pigs [60–62]. Furthermore, partially replacing SBM in a diet containing 118 g/kg of SBM with ESBM at 40 g/kg increased body weight gain, ileal villus height, and tight junction protein expression of the small intestine in nursery pigs [63]. Second, zinc oxide in the diets for phases 1 and 2 might have masked the beneficial effects of replacing SBM with the processed SBM. When undigested glycinin and β-conglycinin from SBM reach the small intestine, they induce excessive oxidative stress, which activates nucleotide-binding oligomerization domain, leucine-rich repeat, and pyrin domain-containing protein 3 (NLRP-3) in pigs [64]. Afterwards, NLRP-3 stimulates caspase-1 activity, which triggers excessive inflammation by activating pro-inflammatory cytokines. The excessive oxidative stress and inflammation damage villus height and tight junction proteins, leading to reduced growth performance in pigs. In contrast, the supplementation of zinc at pharmacological level in the form of zinc oxide in nursery diets can potentially increase the expression of metallothioneins (e.g., superoxide dismutase), which have antioxidant properties due to their metal-binding ability [65]. Furthermore, zinc oxide has the potential to improve adaptive immunity and suppress the transcription of pro-inflammatory cytokines and increase the expression of anti-inflammatory cytokines both locally and systemically. In addition to its antioxidant and anti-inflammatory properties, zinc oxide exhibits antimicrobial effects against pathogenic bacteria responsible for post-weaning diarrhea by suppressing their growth [66]. Therefore, zinc oxide might have maksed the beneficial effects of partially replacing SBM with processed SBM on growth performance and immune status of nursery pigs based on the aforementioned mechanisms. However, the authors should note that serum (d 24) and jejunum samples (d 27) were collected near the end of the experiment. Ma et al [16] reported that a partial replacement of SBM with ESBM decreased the concentration of malondialdehyde (indicator of oxidation of fat) in serum of nursery pigs on d 14 post-weaning, but no difference was observed on d 28 post-weaning. This result indicates that the effects of a partial replacement of SBM with processed SBM may be more pronounces when nursery pigs are younger, which requires experimental validation.

In conclusion, a partial replacement of SBM with ESBM at 70 g/kg increased gain to feed ratio from d 5 to 15, whereas a partial replacement of SBM with PFSBM at 70 g/kg increased the relative abundance of *Lactobacillus* and decreased the abundance of *Pseudomonas* in the jejunal mucosa. However, partially replacing SBM with various processed SBM did not affect immune responses, intestinal morphology, and overall growth performance, which might have been masked by the use of zinc at pharmacological level in early phases.

## Figures and Tables

**Table 1:** Chemical composition and microbial population of soybean meal (SBM), enzyme-treated soybean meal (ESBM), fermented soybean meal (FSBM), and fermented soybean meal containing probiotic microorganism (PFSBM)

Item (as-is basis)	SBM	ESBM	FSBM	PFSBM
Dry matter (g/kg)	899	920	971	953
Crude protein (g/kg)	470	560	422	568
Indispensable amino acids (g/kg)				
Arginine	28.4	36.2	32.9	31.7
Histidine	11.1	14.3	14.6	13.0
Isoleucine	20.4	25.1	25.2	25.3
Leucine	33.8	41.5	42.0	41.2
Lysine	29.8	33.9	34.0	33.2
Methionine	6.6	2.1	2.4	2.4
Phenylalanine	22.2	27.1	29.3	27.0
Threonine	21.2	35.4	31.1	30.6
Tryptophan	5.3	6.6	6.6	6.9
Valine	22.5	26.3	26.3	27.3
*Bacillus amyloliquefaciens* population (CFU/g)	-	-	1.1×10^2^	1.9×10^7^

**Table 2:** Molecular weight distribution of peptides in soybean meal (SBM), enzyme-treated soybean meal (ESBM), fermented soybean meal (FSBM), and fermented soybean meal containing probiotic microorganism (PFSBM) measured by analytical ultracentrifuge

Item	Molecular weight (Da)	Percentage (%)	Average molecular weight (Da)
SBM	134,000	12.9	34,300
	30,100	55.6	
	883	31.5	
ESBM	61,015	2.1	13,747
	14,700	83.9	
	943	14	
FSBM	10,750	16.4	2,816
	1,259	83.6	
PFSBM	7,870	75.6	6,057
	440	24.4	

**Table 3:** Ingredient composition of the experimental diets containing soybean meal (SBM), enzyme-treated soybean meal (ESBM), fermented soybean meal (FSBM), or fermented soybean meal containing probiotic microorganism (PFSBM)

Ingredient (g/kg)	Phase 1 (d 0 to 5)				Phase 2 (d 5 to 15)				Phase 3 (d 15 to 27)			
		
	SBM	ESBM	FSBM	PFSBM	SBM	ESBM	FSBM	PFSBM	SBM	ESBM	FSBM	PFSBM
Corn	402.2	415.6	415.7	415.7	552.1	563.9	564.0	564.0	667.8	680.2	680.4	680.4
Whey permeate	220.0	220.0	220.0	220.0	100.0	100.0	100.0	100.0	-	-	-	-
Poultry meal	70.0	70.0	70.0	70.0	30.0	30.0	30.0	30.0	-	-	-	-
Blood plasma	60.0	60.0	60.0	60.0	30.0	30.0	30.0	30.0	-	-	-	-
SBM	200.0	123.0	123.0	123.0	230.0	153.0	153.0	153.0	280.0	203.0	203.0	203.0
ESBM	-	70.0	-	-	-	70.0	-	-	-	70.0	-	-
FSBM	-	-	70.0	-	-	-	70.0	-	-	-	70.0	-
PFSBM	-	-	-	70.0	-	-	-	70.0	-	-	-	70.0
Poultry fat	20.0	14.0	14.0	14.0	25.0	20.0	20.0	20.0	20.0	15.0	15.0	15.0
L-Lysine HCl	5.0	4.9	4.9	4.9	5.0	5.0	5.0	5.0	4.7	4.6	4.6	4.6
DL-Methionine	2.3	2.2	2.1	2.1	1.9	1.8	1.7	1.7	1.5	1.4	1.3	1.3
L-Threonine	1.5	1.3	1.3	1.3	1.5	1.3	1.3	1.3	1.5	1.3	1.2	1.2
Dicalcium phosphate	4.0	4.0	4.0	4.0	9.0	9.0	9.0	9.0	11.5	11.5	11.5	11.5
Limestone	8.5	8.5	8.5	8.5	9.0	9.5	9.5	9.5	9.0	9.0	9.0	9.0
Vitamin premix^1)^	0.3	0.3	0.3	0.3	0.3	0.3	0.3	0.3	0.3	0.3	0.3	0.3
Mineral premix^2)^	1.5	1.5	1.5	1.5	1.5	1.5	1.5	1.5	1.5	1.5	1.5	1.5
Salt	2.2	2.2	2.2	2.2	2.2	2.2	2.2	2.2	2.2	2.2	2.2	2.2
Zinc oxide	2.5	2.5	2.5	2.5	2.5	2.5	2.5	2.5	-	-	-	-

1) The vitamin premix provided the following per kilogram diet: 3,968 IU of vitamin A; 1,190 IU of vitamin D_3_; 20 IU of vitamin E; 0.012 mg of vitamin B_12_; 4.0 mg of riboflavin; 33 mg of niacin; 6.6 mg of d-pantothenic acid; 1.2 mg of menadione; 0.012 IU of biotin.

2) The mineral premix provided the following per kilogram diet: 17 mg of Cu; 0.297 mg of I; 110 mg of Fe; 33 mg of Mn; 0.297 mg of Se; 110 mg of Zn.

**Table 4:** Nutritional composition of the experimental diets containing soybean meal (SBM), enzyme-treated soybean meal (ESBM), fermented soybean meal (FSBM), or fermented soybean meal containing probiotic microorganism (PFSBM)

Item (as-fed basis)	Phase 1 (d 0 to 5)				Phase 2 (d 5 to 15)				Phase 3 (d 15 to 27)			
		
	SBM	ESBM	FSBM	PFSBM	SBM	ESBM	FSBM	PFSBM	SBM	ESBM	FSBM	PFSBM
Calculated composition												
Metabolizable energy (kcal/kg)	3,420	3,421	3,423	3,423	3,421	3,425	3,427	3,427	3,386	3,392	3,394	3,394
Crude protein (g/kg)	234	237	237	237	208	211	211	211	195	199	198	198
SID lysine (g/kg)	15.0	15.0	15.0	15.0	13.5	13.5	13.5	13.5	12.3	12.3	12.3	12.3
SID methionine+cysteine (g/kg)	8.2	8.2	8.2	8.2	7.4	7.4	7.4	7.4	6.8	6.8	6.8	6.8
SID threonine (g/kg)	8.8	8.8	8.8	8.8	7.9	7.9	7.9	7.9	7.3	7.3	7.3	7.3
SID tryptophan (g/kg)	2.5	2.5	2.5	2.5	2.2	2.2	2.2	2.2	2.0	2.0	2.0	2.0
SID valine (g/kg)	7.9	8.2	8.3	8.3	7.6	7.9	7.9	7.9	7.5	7.8	7.9	7.9
Calcium (g/kg)	8.5	8.5	8.5	8.5	8.1	8.1	8.1	8.1	7.1	7.1	7.1	7.1
STTD phosphorus (g/kg)	4.5	4.5	4.5	4.5	4.0	4.0	4.0	4.0	3.3	3.3	3.3	3.3
Analyzed composition												
Crude protein (g/kg)	213	219	215	219	194	195	212	192	186	182	181	179

SID, standardized ileal digestible; STTD, standardized total tract digestible.

**Table 5:** Alpha diversity metrics (Chao1, Shannon, and Simpson) of jejunal mucosa-associated microbiota at the genus level of pigs fed diets containing soybean meal (SBM), enzyme-treated soybean meal (ESBM), fermented soybean meal (FSBM), or fermented soybean meal containing probiotic microorganism (PFSBM)^1)^

Item	SBM	ESBM	FSBM	PFSBM	SEM	p-value
Chao1	18.9	12.0	16.9	15.4	2.5	0.319
Shannon	1.39	0.95	1.28	1.23	0.15	0.215
Simpson	0.52	0.38	0.51	0.48	0.05	0.232

1) Each least squares mean represents 12 observations.

SEM, standard error of the mean.

**Table 6:** Relative abundance of jejunal mucosa-associated microbiota at the phylum level of pigs fed diets containing soybean meal (SBM), enzyme-treated soybean meal (ESBM), fermented soybean meal (FSBM), or fermented soybean meal containing probiotic microorganism (PFSBM)^1)^

Item	SBM	ESBM	FSBM	PFSBM	SEM	p-value
*Proteobacteria*	87.2	85.3	75.4	86.7	5.9	0.454
*Firmicutes*	11.5	14.3	22.6	12.3	5.3	0.448
*Bacteroidetes*	0.2	0.3	0.9	0.4	0.4	0.703
*Actinobacteria*	0.3	0.1	0.2	0.3	0.1	0.638
*Chlamydiae*	0.7	0.0	1.0	0.3	0.6	0.579
*Cyanobacteria*	0.0	0.0	0.0	0.1	0.0	0.547

1) Each least squares mean represents 12 observations.

SEM, standard error of the mean.

**Table 7:** Relative abundance of jejunal mucosa-associated microbiota at the family level of pigs fed diets containing soybean meal (SBM), enzyme-treated soybean meal (ESBM), fermented soybean meal (FSBM), or fermented soybean meal containing probiotic microorganism (PFSBM)^1)^

Item	SBM	ESBM	FSBM	PFSBM	SEM	p-value
*Acetobacteraceae*	0.01	0.00	0.01	0.01	0.00	0.611
*Acidaminococcaceae*	0.02	0.00	0.12	0.00	0.06	0.463
*Aeromonadaceae*	0.00	0.00	0.00	0.00	0.00	0.582
*Bacillaceae*	0.06	0.08	0.05	0.07	0.06	0.965
*Bifidobacteriaceae*	0.07	0.05	0.17	0.09	0.08	0.713
*Brachyspiraceae*	0.12	0.00	0.00	0.03	0.06	0.447
*Campylobacteraceae*	0.53	0.05	0.26	0.44	0.17	0.222
*Carnobacteriaceae*	0.02	0.01	0.02	0.00	0.01	0.334
*Caulobacteraceae*	0.10	0.00	0.02	0.02	0.05	0.518
*Chlamydiaceae*	0.74	0.00	0.27	1.00	0.60	0.579
*Clostridiaceae*	1.08	3.87	0.81	2.08	2.18	0.799
*Coriobacteriaceae*	0.00	0.00	0.01	0.00	0.00	0.288
*Corynebacteriaceae*	0.00	0.07	0.10	0.09	0.10	0.943
*Enterobacteriaceae*	0.00	0.02	0.18	0.03	0.09	0.468
*Erysipelotrichaceae*	0.57	0.00	0.00	0.00	0.29	0.415
*Eubacteriaceae*	0.04	0.00	0.01	0.03	0.03	0.675
*Helicobacteraceae*	22.98	8.76	22.80	32.96	12.15	0.524
*Lachnospiraceae*	0.04	0.09	0.00	0.13	0.08	0.648
*Lactobacillaceae*	1.07^a^	0.96^a^	0.96^a^	12.35^b^	3.31	0.033
*Moraxellaceae*	0.01	0.02	0.09	0.07	0.06	0.613
*Nostocaceae*	0.03	0.03	0.05	0.02	0.03	0.860
*Oxalobacteraceae*	0.02	0.00	0.00	0.12	0.06	0.439
*Pasteurellaceae*	2.16	0.11	0.00	0.03	1.10	0.430
*Peptostreptococcaceae*	0.15	0.02	0.00	1.26	0.65	0.442
*Porphyromonadaceae*	0.00	0.00	0.02	0.12	0.06	0.447
*Prevotellaceae*	0.21	0.18	0.40	0.71	0.37	0.712
*Propionibacteriaceae*	0.10	0.02	0.01	0.00	0.03	0.167
*Pseudomonadaceae*	53.28	61.43	51.96	33.90	8.80	0.135
*Rhodobacteraceae*	0.00	0.01	0.02	0.00	0.01	0.238
*Rikenellaceae*	0.00	0.09	0.00	0.00	0.04	0.413
*Ruminococcaceae*	0.00	0.29	1.61	0.01	0.81	0.455
*Staphylococcaceae*	6.81	8.62	7.65	4.57	1.19	0.120
*Streptococcaceae*	0.07	0.00	0.06	0.04	0.03	0.353
*Succinivibrionaceae*	0.10	0.06	0.04	0.32	0.17	0.613
*Veillonellaceae*	0.68	0.31	1.02	1.49	0.83	0.774
*Xanthomonadaceae*	8.51	14.84	11.28	7.91	2.67	0.105

1) Each least squares mean represents 12 observations.

a,b Least squares means without a common lowercase superscript differ (p<0.05).

SEM, standard error of the mean.

**Table 8:** Relative abundance of jejunal mucosa-associated microbiota at the genus level of pigs fed diets containing soybean meal (SBM), enzyme-treated soybean meal (ESBM), fermented soybean meal (FSBM), or fermented soybean meal containing probiotic microorganism (PFSBM)^1)^

Item	SBM	ESBM	FSBM	PFSBM	SEM	p-value
*Pseudomonas*	55.61^b^	47.58^ab^	54.00^b^	30.84^a^	10.56	0.072
*Helicobacter*	19.30	8.64	22.78	32.83	11.58	0.520
*Staphylococcus*	6.53	7.21	7.53	4.53	1.18	0.126
*Lactobacillus*	1.07^a^	0.55^a^	0.80^a^	12.35^b^	3.35	0.031
*Candidatus arthromitus*	0.24	4.48	0.08	0.09	1.75	0.400
*Clostridium*	1.74	0.02	0.18	1.90	1.27	0.525
*Bacillus*	2.09	0.04	0.05	0.57	1.11	0.472
*Stenotrophomonas*	0.61^ab^	0.82^a^	0.68^ab^	0.36^b^	0.27	0.037
*Chlamydia*	0.74	0.03	0.27	1.00	0.60	0.579
*Actinobacillus*	1.84	0.12	0.00	0.03	0.93	0.431
*Prevotella*	0.18	0.16	0.33	0.62	0.32	0.707
*Campylobacter*	0.51	0.04	0.26	0.42	0.17	0.232
*Mitsuokella*	0.26	0.14	0.25	0.56	0.27	0.757
*Dialister*	0.01	0.02	0.47	0.15	0.24	0.493
*Turicibacter*	0.57	0.00	0.00	0.00	0.28	0.415
*Megasphaera*	0.04	0.06	0.16	0.21	0.10	0.603
*Succinivibrio*	0.05	0.04	0.03	0.32	0.16	0.527
*Bifidobacterium*	0.07	0.06	0.17	0.09	0.08	0.717
*Corynebacterium*	0.11	0.07	0.03	0.09	0.09	0.617
*Streptococcus*	0.07	0.00	0.06	0.04	0.03	0.343
*Faecalibacterium*	0.00	0.15	0.00	0.01	0.06	0.424

1) Each least squares mean represents 12 observations.

a,b Least squares means without a common lowercase superscript differ (p<0.05).

A,B Least squares means without a common uppercase superscript tend to be different (0.05≤p<0.10).

SEM, standard error of the mean.

**Table 9:** Immune responses from jejunal mucosa of pigs fed diets containing soybean meal (SBM), enzyme-treated soybean meal (ESBM), fermented soybean meal (FSBM), or fermented soybean meal containing probiotic microorganism (PFSBM)^1)^

Item	SBM	ESBM	FSBM	PFSBM	SEM	p-value
IgA						
Jejunum (μg/mg protein)	0.9	1.3	1.0	1.1	0.2	0.264
IgG						
Serum (mg/mL)	2.4	1.9	1.8	2.0	0.2	0.202
Jejunum (uμg/mg protein)	1.4	1.1	1.2	1.3	0.3	0.729
TNF-α						
Serum (mg/mL)	125^ab^	93^a^	114^ab^	142^b^	18	0.063
Jejunum (uμg/mg protein)	1.8	1.7	1.8	2.1	0.2	0.448
IL-6						
Serum (mg/mL)	14.4	11.3	11.6	17.3	2.6	0.131
Jejunum (uμg/mg protein)	1.6	1.3	1.5	1.4	0.2	0.197

1) Each least squares mean represents 12 observations.

A,B Least squares means without a common uppercase superscript tend to be different (0.05≤p<0.10).

SEM, standard error of the mean.

**Table 10:** Jejunal morphology of pigs fed diets containing soybean meal (SBM), enzyme-treated soybean meal (ESBM), fermented soybean meal (FSBM), or fermented soybean meal containing probiotic microorganism (PFSBM)^1)^

Item	SBM	ESBM	FSBM	PFSBM	SEM	p-value
Villus height (μm)	361	362	358	388	18	0.778
Crypt depth (μm)	122	150	137	152	28	0.439
Villus height to crypt depth ratio	3.4	2.8	3.0	3.0	0.6	0.630

1) Each least squares mean represents 12 observations.

SEM, standard error of the mean.

**Table 11:** Growth performance of pigs fed diets containing soybean meal (SBM), enzyme-treated soybean meal (ESBM), fermented soybean meal (FSBM), or fermented soybean meal containing probiotic microorganism (PFSBM)^1)^

Item^3^	SBM	ESBM	FSBM	PFSBM	SEM	p-value
Body weight (kg)						
d 0	7.4	7.5	7.4	7.4	0.7	0.923
d 5	8.7	8.8	8.9	8.7	1.0	0.875
d 15	11.6	12.8	12.6	11.8	1.5	0.300
d 27	17.7	19.9	18.9	18.0	2.0	0.212
Average daily gain (g/d)						
Phase 1 (d 0 to 5)	263	260	302	257	65	0.838
Phase 2 (d 5 to 15)	290	406	364	312	51	0.111
Phase 3 (d 15 to 27)	502	590	524	516	43	0.185
Overall (d 0 to 27)	379	461	424	392	50	0.227
Average daily feed intake (g/d)						
Phase 1 (d 0 to 5)	325	359	371	308	53	0.657
Phase 2 (d 5 to 15)	582	603	583	522	63	0.516
Phase 3 (d 15 to 27)	825	930	852	800	57	0.115
Overall (d 0 to 27)	632	703	663	606	59	0.262
Gain to feed ratio (g/g)						
Phase 1 (d 0 to 5)	0.857	0.686	0.814	0.778	0.065	0.268
Phase 2 (d 5 to 15)	0.487^a^	0.668^b^	0.596^ab^	0.590^ab^	0.042	0.029
Phase 3 (d 15 to 27)	0.602	0.633	0.610	0.645	0.024	0.244
Overall (d 0 to 27)	0.588	0.652	0.627	0.642	0.030	0.108

1) Each least squares mean represents 12 observations.

a,b Least squares means without a common lowercase superscript differ (p<0.05).

SEM, standard error of the mean.
